# Population and Genetic Study of *Vibrio cholerae* from the Amazon Environment Confirms that the *WASA-1* Prophage Is the Main Marker of the Epidemic Strain that Circulated in the Region 

**DOI:** 10.1371/journal.pone.0081372

**Published:** 2013-11-26

**Authors:** Lena Líllian Canto de Sá Morais, Daniel Rios Garza, Edvaldo Carlos Brito Loureiro, Elivam Rodrigues Vale, Denise Suéllem Amorim de Sousa Santos, Vanessa Cavaleiro Corrêa, Nayara Rufino Sousa, Tereza Cristina Monteiro Gurjão, Elisabeth Conceição de Oliveira Santos, Verônica Viana Vieira, Erica Lourenço da Fonseca, Ana Carolina Paulo Vicente

**Affiliations:** 1 Environmental Section of the Evandro Chagas Institute, Ananindeua, Pará, Brazil; 2 Bacteriology Section of the Evandro Chagas Institute, Ananindeua, Pará, Brazil; 3 Laboratory of Molecular Genetics of Microorganisms, Oswaldo Cruz Foundation, Rio de Janeiro, Rio de Janeiro, Brazil; Radboud University Medical Centre, NCMLS, Netherlands

## Abstract

*Vibrio cholerae* is a natural inhabitant of many aquatic environments in the world. Biotypes harboring similar virulence-related gene clusters are the causative agents of epidemic cholera, but the majority of strains are harmless to humans. Since 1971, environmental surveillance for potentially pathogenic *V. cholerae* has resulted in the isolation of many strains from the Brazilian Amazon aquatic ecosystem. Most of these strains are from the non-O1/non-O139 serogroups (NAGs), but toxigenic O1 strains were isolated during the Latin America cholera epidemic in the region (1991-1996). A collection of environmental *V. cholerae* strains from the Brazilian Amazon belonging to pre-epidemic (1977-1990), epidemic (1991-1996), and post-epidemic (1996-2007) periods in the region, was analyzed. The presence of genes related to virulence within the species and the genetic relationship among the strains were studied. These variables and the information available concerning the strains were used to build a Bayesian multivariate dependency model to distinguish the importance of each variable in determining the others. Some genes related to the epidemic strains were found in environmental NAGs during and after the epidemic. Significant diversity among the virulence-related gene content was observed among O1 strains isolated from the environment during the epidemic period, but not from clinical isolates, which were analyzed as controls. Despite this diversity, these strains exhibited similar PFGE profiles. PFGE profiles were significant while separating potentially epidemic clones from indigenous strains. No significant correlation with isolation source, place or period was observed. The presence of the *WASA-1* prophage significantly correlated with serogroups, PFGE profiles, and the presence of virulence-related genes. This study provides a broad characterization of the environmental *V. cholerae* population from the Amazon, and also highlights the importance of identifying precisely defined genetic markers such as the *WASA-1* prophage for the surveillance of cholera.

## Introduction

*Vibrio cholerae* is a genetically diverse inhabitant of the aquatic environment. Its lifestyle is intimately associated to the micro-fauna of brackish and estuarine waters, where it can be found as a free-living microbe or in diverse associations with zooplankton, phytoplankton, and other species [1-4]. Such diverse habitats in preferably warm and slightly alkaline environments constitute the species’ natural reservoir [5]. Several studies have shown that great genetic diversity is observed at the intraspecific level [6,7]. This diversity reflects the dynamic existence of many *V. cholerae* populations in diverse aquatic environments throughout the world.

Pathogenic *V. cholerae* are also diverse groups of strains with relatively distinct genomic backgrounds. Nevertheless, epidemic and pandemic cholera belong to a few clusters of relatively clonal populations of independent origin [8,9]. Evolutionary studies that consider the complete genome sequences or housekeeping genes of *V. cholerae* strains generally agree that the seventh and ongoing cholera pandemic—which constitutes one of these population-clusters—originated from a lineage that can be distinctly identified [8,10,11]. This lineage is characterized by: (i) the O1 surface antigen; (ii) several gene systems which are related to the ability to colonize the human intestine, thus coordinately expressing virulence factors, and provoking cholera; and (iii) a number of genetic and physiological properties which define a biotype named El Tor [12]. In all of the outbreaks attributed to the seventh cholera pandemic, toxigenic O1 El Tor strains have been isolated from stool and, frequently, from the environment. This lineage is distinguishable through several typing methods from indigenous strains that are majorly non-toxigenic and of NAG serogroups [13-16].

The seventh cholera pandemic emerged in 1961 and since then its causative agent—the toxigenic O1 El Tor *V. cholerae*—has become endemic in areas of Africa, Asia and Latin America [17-19]. Epidemic outbreaks in endemic regions frequently exhibit periodicity that coincides with environmental variables such as yearly monsoons, tidal estuaries, and riverine systems [20-22]. Outbreaks of cholera in areas where it is not endemic are usually attributed to importation, while the extent of the epidemics in both endemic and epidemic settings are related to human transportation routes and risk factors encountered in the places where cholera reaches [23,24]. Poverty, malnutrition, crowding, migration, and poor sanitation, among other social and economic drivers, have been correlated to the extent and fatality-rate of cholera outbreaks. Other factors such as climate change and natural disasters also seem to play an important role in favoring the emergence and spread of cholera [25-27]. 

The origin of the last cholera epidemic in South America that started in Peru (1991) has not been completely elucidated. In this epidemic, a distinguishable toxigenic O1 El Tor clone was predominantly isolated from confirmed cholera cases. This clone exhibited important variations compared to other toxigenic O1 El Tor strains that were being isolated in other regions of the world [28]. Among these differences, the presence of a 50 kb prophage integrated into the alanine aminopeptidase gene was recently identified [8,29,30]. This prophage has been named *WASA-1*. 

Before the introduction of cholera in 1991, environmental surveillance for the presence of potentially toxigenic *V. cholerae* strains was initiated in several Latin American countries. These initiatives followed the eminent risk of importation of the disease from countries that experienced cholera outbreaks [31]. Many environmental isolates were recovered in this period, including non-toxigenic O1s [32,33]. In the Brazilian Amazon, surveillance of wastewater in the city of Belem also isolated many *V. cholerae* NAG strains. During and after the epidemic, more extensive environmental surveillance has shown the presence of NAG strains in many rivers and streams, and toxigenic O1 isolates were also found during the epidemic period. Though the toxigenic O1 El Tor *V. cholerae* has failed to establish long-term endemicity in the Amazon, several potential risk factors, compared to the dynamics of cholera in other regions, highlight the need for continuous and effective surveillance of the bacterium’s natural reservoir. Some of these factors are: (i) the presence of endemic populations of *V. cholerae* in many of the region’s rivers and streams, which can undergo lateral gene transfer and constitute a natural reservoir of virulence genes [34]; (ii) an extensive human population that occupies water margins in both urban and rural areas, which lack appropriate sanitary infrastructure; (iii) periodic flooding of urban neighborhoods during the yearly rainy season; (iv) many sites of balneability and recreation that are potential sources of water contamination and of consumption of contaminated water; and (v) chronic inadequate supply of clean water even in urbanized areas, which represent high risk of fecal contamination.

The epidemiological importance of the Amazon region as a potential border for cholera in Brazil has been clearly indicated in two independent historical periods. During the third pandemic, cholera was introduced by a “caravella” coming from Europe into the country through the Amazon region. This epidemic extensively struck Brazil, infecting approximately 200 thousand people in 1855 [35]. Later, in the seventh pandemic, cholera again entered Brazil through the west-Amazon by the Peruvian border [36]. In this epidemic, the disease exhibited temporary endemicity with seasonal peaks for approximately six years [37]. The spread of cholera to other regions of the country in both of these occasions indicates the importance of the region and its environment concerning the risk of cholera in Brazil. This also indicates the importance of characterizing and monitoring the dynamic *V. cholerae* population in the Amazon environment.

The purpose of this study was to analyze a collection of environmental *V. cholerae* strains from the Amazon, spanning a large historical period which can roughly be divided into pre-epidemic (1977-1990), epidemic (1991-1996), and post-epidemic (1996-2007) periods. The presence of genes related to virulence phenotypes within the species was surveyed and the genetic relationship among the strains was analyzed through genomic restriction patterns. Using these variables and the information available concerning the strains, a Bayesian multivariate dependency model was employed to distinguish the importance of each variable on determining the others. Besides the broad characterization of the environmental *V. cholerae* population of the Amazon, this study reinforces the importance of identifying precisely defined genetic markers such as the *WASA-1* prophage for the surveillance of cholera. 

## Materials and Methods

### *V. cholerae* strains and growth conditions

**Figure 1:**
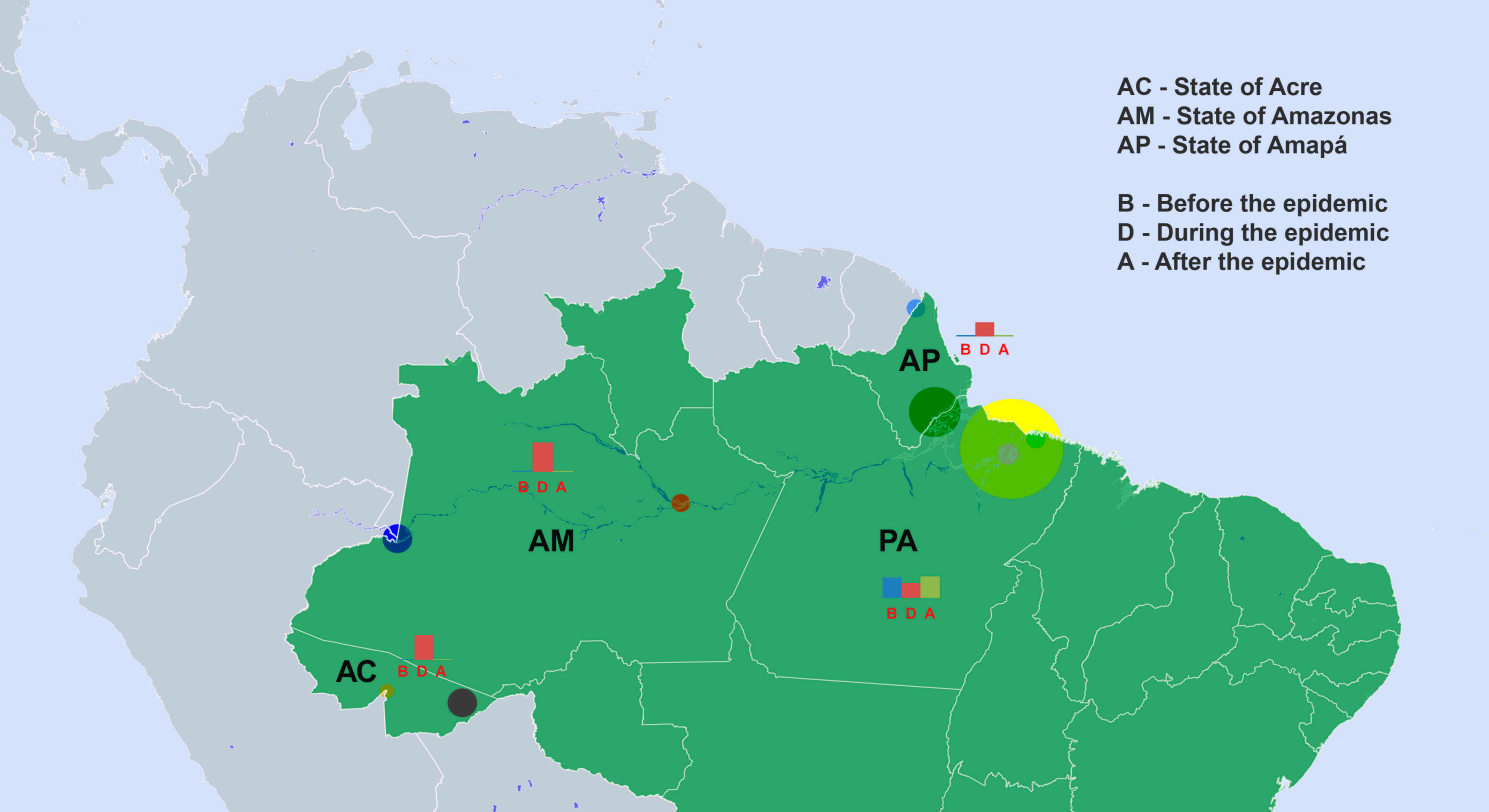
Geographical distribution of *V. cholerae* isolates. The geographical location of rivers, streams, and wastewater plants from where the strains that were used in this study were isolated are indicated in the map. The sizes of markers indicate the number of strains in each location, markers are centered in the cities where the strains were isolated (see Table S1). Belem (yellow), Barcarena (light green), Maruda (pink), Macapá (dark green), Oiapoque (light blue), Manaus (red), Tabatinga (light blue), Rio Branco (purple), and Santa Rosa (orange). Quantities of strains isolated in each period are indicated in the bar graphs.

A total of 125 *V. cholerae* strains that were isolated from the environment at different sites in the Brazilian Amazon in the period of 1977 to 2007 were analyzed (Figure **1** and Table **1**). Four strains from stool samples of patients that were isolated in 1991-1994 during the Amazon cholera epidemic were also included as references; one clinical strain from a cholera-like outbreak that occurred in the Amazon in 1992 and was caused by a non-toxigenic O1 strain was also included; a classical strain isolated in Pakistan, 1986, and two Asian El Tor strains from India and Bangladesh, isolated in 1971 and 1973, respectively, were also used as references (Table **S1**). 

**Table 1:** Environmental strains of *V. cholerae* isolated from the Amazon environment.

	**Pre-epidemic**	**Epidemic**	**Post-epidemic**	**Total**
**Number of strains**	33	62	35	130
**Serogroup (NAGs)**	33	37	34	104
**Serogroup (O1s)**	0	25	1	26
**Source (Copepod)**	0	0	2	2
**Source (Fish)**	0	3	0	3
**Source (Stool)**	0	5	0	5
**Source (River)**	0	29	20	49
**Source (Stream)**	0	9	10	19
**Source (Wastewater)**	33	16	3	54
**State (Pará)**	33	24	35	94
**State (Amapá)**	0	23	0	23
**State (Acre)**	0	7	0	7
**State (Amazonas)**	0	8	0	8

All strains were from the bacterial collection of the Evandro Chagas Institute, Brazil, and were re-isolated and characterized following standard procedures for *V. cholerae*. Briefly, strains were cultured in alkaline peptone water (APW) and plated on thiosulphate citrate bile salt sucrose (TCBS). Colonies were further examined by biochemical assays, which included: oxidase; motility; fermentation on glucose, sucrose, and lactose; growth in 0% NaCl; lysine decarboxylase, arginine dehydrolase, ornithine decarboxylase, methyl-red, Voges-Proskauer, and indole tests. Serogouping was performed with a polyvalent O1 and O139 antisera, using the slide agglutination test (Mast Diagnostics Ltd). Species identification was further confirmed by PCR using primers for the species-specific gene *ompW* according to Nandi et al.(2000) [38]. 

### PCR Assays

Total genomic DNA was purified as previously described [39]. PCR assays were performed in all strains using specific primers for all genes listed in Table **2**. Platinum TAQ kit (Invitrogen) was used for DNA amplification following manufacturer’s instructions. Specific annealing temperatures were used for each primer and results were assessed through electrophoresis (for primer sequences and annealing temperatures, see Table **S2**).

**Table 2:** Genes investigated by PCR.

**Gene**	Abbrev.	Locus Tag	Ref. Genome (Ac. Number)
Cholera enterotoxin subunit A	*ctxA*	VC1456	N16961 (NC_002505.1)
Cholera enterotoxin subunit B	*ctxB*	VC1457	N16961 (NC_002505.1)
Zonula occludens toxin	*zot*	VC1458	N16961 (NC_002505.1)
Accessory cholera enterotoxin	*ace*	VC1459	N16961 (NC_002505.1)
Hypothetical protein	*orfU*	VC1460	N16961 (NC_002505.1)
RTX toxin A	*rtxA*	VC1451	N16961 (NC_002505.1)
RTX toxin activating protein	*rtxC*	VC1450	N16961 (NC_002505.1)
Cholera toxin transcriptional activator	*toxR*	VC0984	N16961 (NC_002505.1)
toxin co-regulated pilin	*tcp*	VC0828	N16961 (NC_002505.1)
Phage replication protein Cri	*ptcl*	VC1469	N16961 (NC_002505.1)
Heat-stable enterotoxin	*stn/sto*	M85198.1	M85198.1
Haemolysin	*hlyA*	VCA0219	N16961 (NC_002506.1)
Lactonizing lipase	*hlyC*	VCA0021	N16961 (NC_002506.1)
Hemolysin secretion protein	*hlyB*	VCA0220	N16961 (NC_002506.1)
Phage deoxyribonucleoside kinase	*WASA-1*	O3Y_07175	IEC224 (NC_016944.1)
Phage glycosyl hydrolase	*WASA-1*	O3Y_07005	IEC224 (NC_016944.1)
Phage RNA polymerase	*WASA-1*	O3Y_07235	IEC224 (NC_016944.1)

### Pulsed-Field Gel Electrophoresis (PFGE)

PFGE was performed according to PulseNet standardized protocol [40] with NotI restriction enzyme (Invitrogen). Plugs were submitted to electrophoresis using the CHEF-DR III equipment (BioRad). In each assay the Lambda PFGE marker (Biolabs) was used. Images of the pulsetypes were uploaded and analyzed using the BioNumerics software, Version 5.1 (Applied Maths). Profiles were compared using the Dice coefficient and clustered by the unweighted pair group method with arithmetic mean (UPGMA). Cluster confidence was assessed by bootstrap with 10^6^ replicates. Clusters were visualized and represented using the Fig Tree software, version 1.4 (tree.bio.ed.ac.uk/software/figtree/). A cladogram representation of the PFGE profiles, as well as images of representative gels can be found in Figure **S1**.

### Multivariate dependency model

Possible interrelations between the multivariate sets of discrete data which were available for each strain (presence of virulence-associated genes, isolation period, source, serogroup, city, and PFGE profiles) were investigated using a statistical learning approach, which is based on the construction of an optimal Bayesian Network (BN) [41]. For this purpose we used the open source internet service, B-Course (http://b-course.hiit.fi/bene) [42]. In this approach, the model was defined from the conditional probabilities (represented as arcs) between variables (represented as nodes), in which a best network was determined by iterating over different topologies and using an expectation maximization algorithm. A precise description of the algorithm implemented in B-Course is available in reference [43]. The strength of each arc is represented as the posterior probability of the model when the edge is removed. 

## Results

### Gene content PCR profiles

The presence of 17 genes was investigated in all isolates (Table **2**). These included several genes related to virulence phenotypes in *V. cholerae*, such as: genes encoded by the genome of the CTXφ bacteriophage (*ctxAB*, *ace*, *zot*, and *orfU*) [44]; *tcpA*, which is the structural component of the toxin corregulated pilus [45]; *toxR*, a major regulator of many genes related to cellular functions, among which are the *V. cholerae* virulence program [46,47]; and the *WASA-1* prophage, a distinguished marker of the Latin American epidemic clone [30]. Other genes that are related to virulence phenotypes and genomic integration of virulence-related elements were also investigated (Table **2**).

**Figure 2:**
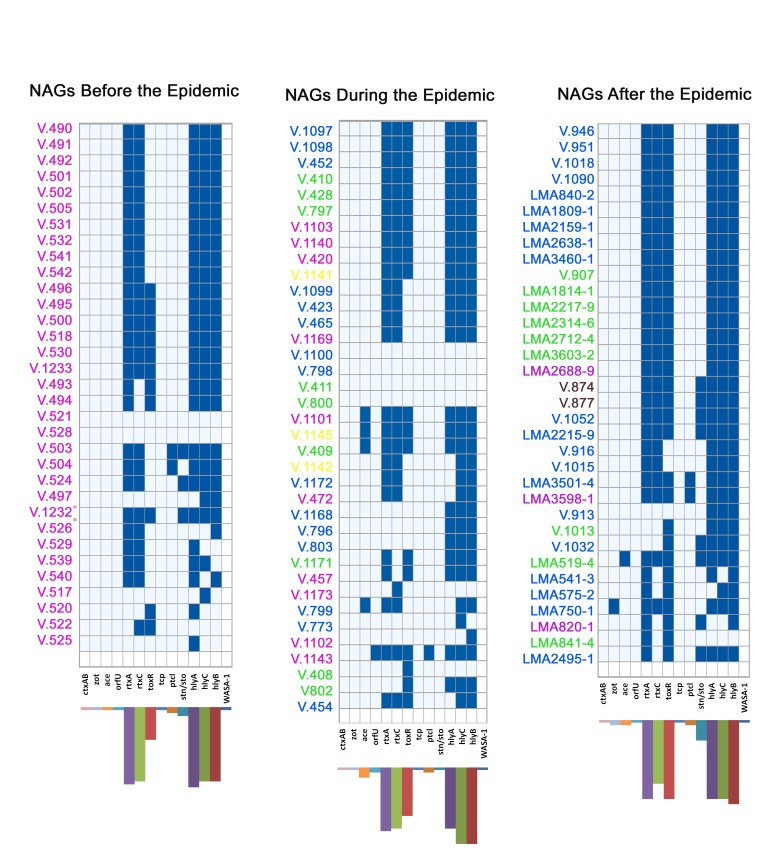
Distribution of genotypes among NAG strains. The presence or absence of virulence-related genes are represented, respectively, by blue and white squares. The histogram below each figure correspond to the frequency of each gene. The colors highlighting the strains’ keys correspond to the isolation sources. Strains highlighted pink were isolated from wastewater, blue from superficial water, green from superficial stream water, yellow from fish, and brown from copepods.

Thirty-five different profiles (genotypes) were observed among the 104 NAG strains. The most frequent profile, present in 30.76% of the strains, was *rtxA, rtxC, toxR, hlyA, hlyC*, and *hlyB*, which were also, individually, the most frequent genes among NAGs of all three periods that were studied (Figure **2**). A temporal pattern was observed in the detection of the the CTXφ genes (Figure **2**). These genes were only found in strains isolated during and after the epidemic period. Nevertheless, none of these genes were co-amplified in any of the NAG strains. No NAG strains harbored *ctxAB*, *tcpA*, or the *WASA-1* prophage genes. The Cri replication protein, which is encoded by the toxin linked cryptic plasmid element (pTLC) exhibited a different pattern: a few strains (2.88%) from all the periods were found to harbor this element which is also reported to be a satellite phage (TLCφ) [48]. Similar to the Cri replication protein, toxR was amplified in strains from all three periods, but seemed to be more frequent among strains isolated during and after the epidemic period. Three strains (9.09%) from before the epidemic period and five strains (14.70%) from the post epidemic period harbored the *stn/sto* toxin gene, while none of the O1 strains were found to have this gene. 

**Figure 3:**
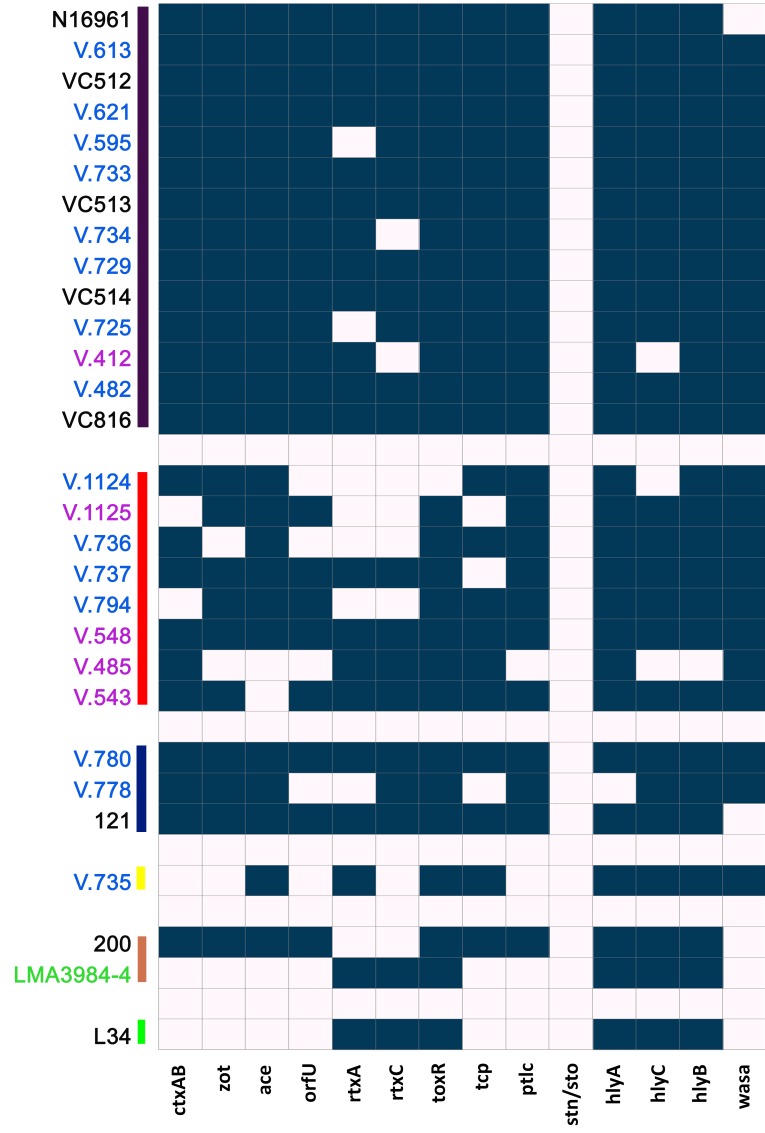
O1 genotypes. The presence and absence of virulence-related genes are represented, respectively, by blue and white squares. The strains are grouped in colored bars according to their PFGE cluster (Fig. 4): from top to bottom are groups 1 (purple), 2 (red), 3 (blue), 9 (yellow), 6 (orange), and 8 (green). The colors highlighting the strain keys correspond to the isolation sources. Strains highlighted pink were isolated from wastewater, blue from superficial water, green from superficial stream water, and black from clinical sources.

**Figure 4:**
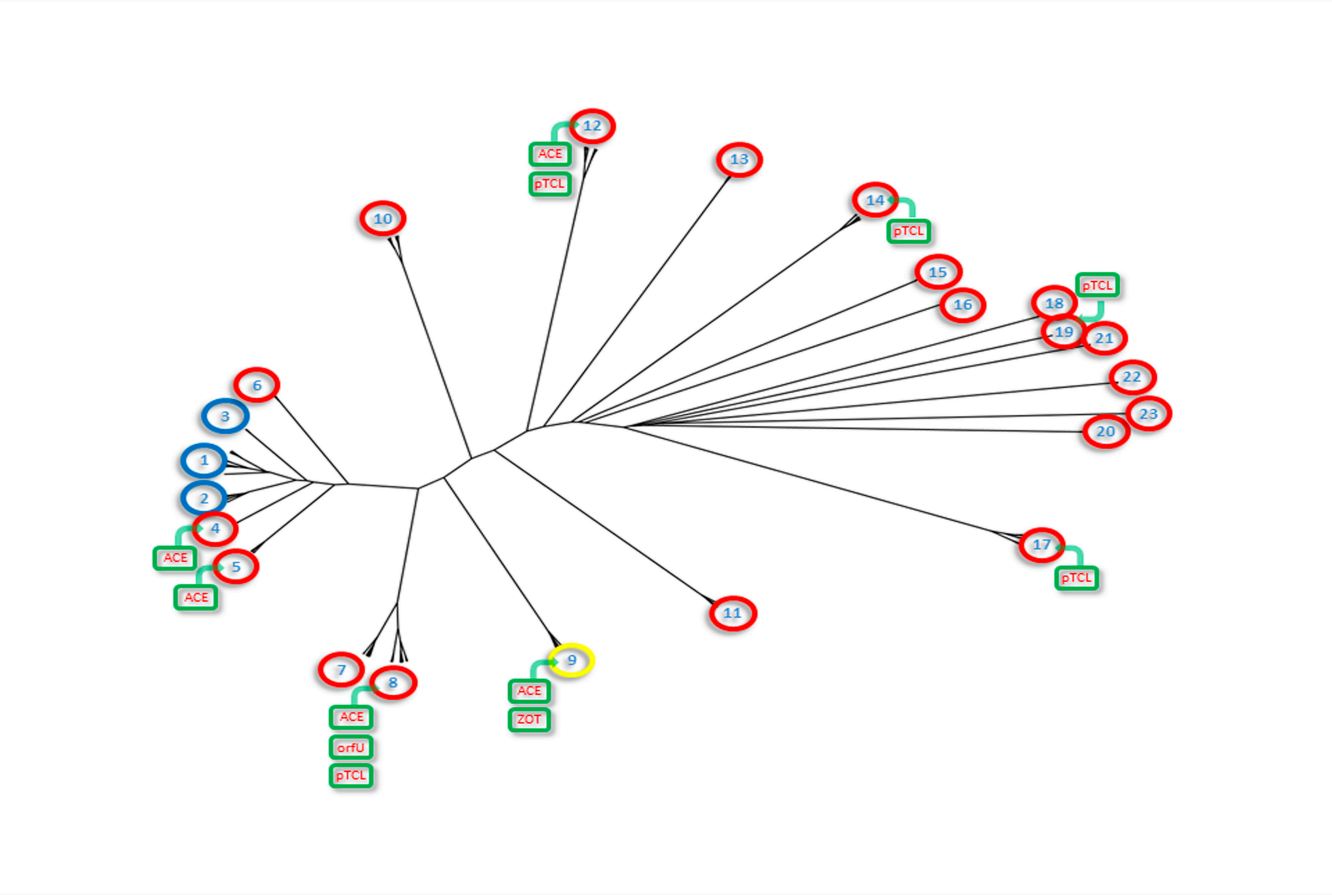
Hierarchical clustering of PFGE profiles. Relative distances are based on the pairwise comparison of band patterns through the dice coefficient and clustering through UPGMA. Blue groups are formed by the epidemic clones, while red groups contain environmental NAGs. The yellow group has one strain (V. 735) which is identified as a possible epidemic clone (see text). The integration of genes which are related to epidemic clones is indicated by squares in each cluster.

Among the O1 strains, all of the clinical El Tor from outbreaks in the Amazon harbored the same set of genes, namely *ctxAB*, *zot*, *ace*, *orfU*, *rtxA*, *rtxC*, *toxR*, *tcpA*, *pTLC*, *hlyA*, *hlyC*, *hlyB*, and the *WASA-1* genes (Figure **3**). The only difference from the clinical El Tor strains from Asia— 121 and N16961—was the presence of the *WASA-1* phage. Diverse virulence related gene contents were observed among the O1 strains that were isolated from the environment but seemed to be related to the epidemic clone. This suggests repeated events of gene loss in the environment. Lack of evidence for the presence of one, two, three, and even four of the genes tested was found in some of these strains, though all of them were positive for the three *WASA-1* prophage genes that were tested. We identified the O1 strains from the PFGE clusters 1, 2, 3, and 9 as being related to the epidemic clone (Figure **4**, discussed below). Among these strains, three of them tested negative for the *ctxAB* genes, while one of these was also negative for *tcpA*. *tcpA* amplification was also absent in two strains that contained *ctxAB* and other genes of the CTXφ. 

The non-toxigenic O1 strain from the Amazonia lineage isolated from cholera-like outbreaks in the Northern Amazon [49] exhibited an identical gene content profile as the also non-toxigenic O1 strain isolated from the Amazon environment in 2007—LMA 3984-4 [50]. Though these strains exhibit identical PCR profiles, their genomic sequences indicate that they´re not clonal [6]. 

### PFGE profiles

Hierarchical clustering analysis of the PFGE profiles defined 23 distinct groups. All of the clinical and environmental O1 El Tor strains from the Amazon were clustered in the groups 1, 2, and 3 (Figure **4**). The N16961 strain was clustered with the strains from the Amazon of group 1, and the strain 121 from India clustered in group 3. The only *WASA-1* positive isolate that did not group within these three clusters was the strain V.735, which clustered within group 9. The LMA 3984-4 strain clustered with the strain 200 of the classical biotype which was isolated in Pakistan, reproducing previously published results [50]. 

**Figure 5:**
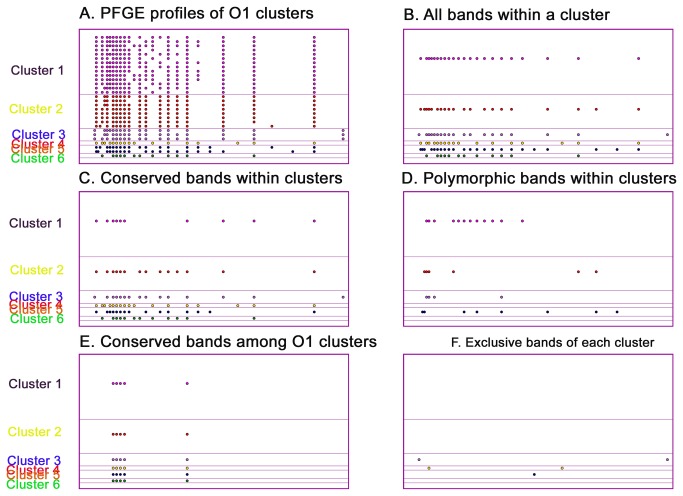
O1 PFGE clusters. Plot of restriction-site band for the six clusters of O1 strains, indicating all bands within a cluster (B), conserved bands within all strains of a cluster(C), bands that vary among strains of the same cluster (D), bands that are conserved among all O1 strains(E), and bands that are unique to any single cluster (F).

A detailed analysis of the PFGE bands that were detected among O1 strains showed that groups 1, 2, and 3 were nearly identical, with only two and three different bands in between them when considering all of the individual cluster’s bands (Figure **5, B**). Greater diversity was observed among polymorphic and conserved bands within each of these clusters (Figure **5** **, C-D**). Five bands were shared among all O1 strains, but only a small number of bands were exclusive of particular clusters, none of which were found in the groups 1 and 2 (Figure **5** **, E-F**). 

**Figure 6:**
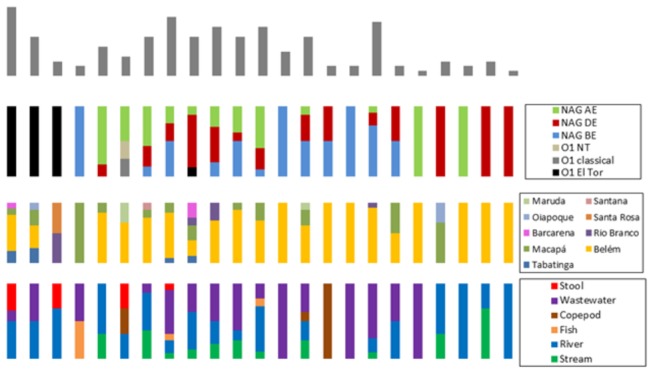
Correlation between PFGE profiles and data from strains. Each column represents a PFGE-profile group. The superior bars indicate the number of strains, while the colors (according to legend) represent the frequency of each of the variables.

Besides the clustering of clinical and environmental O1 El Tor strains, the PFGE profiles were not found to be informative of any other variable investigated in this study. As shown in Figure 4, the virulence genes that were predominant in the toxigenic O1 strains were detected in different clusters among NAG strains (Table **S1**). Additionally, no significant correlation was found between PFGE group, serogroup, period, city of isolation, and source (Figure **6**). In fact, only small groups of strains isolated in similar periods and cities clustered together, such as groups 4, 13, 16, 21, and 22, few of which were isolated from the same environmental sources (Figure **6** and Table **S1**). 

### Multivariate dependency model

**Figure 7:**
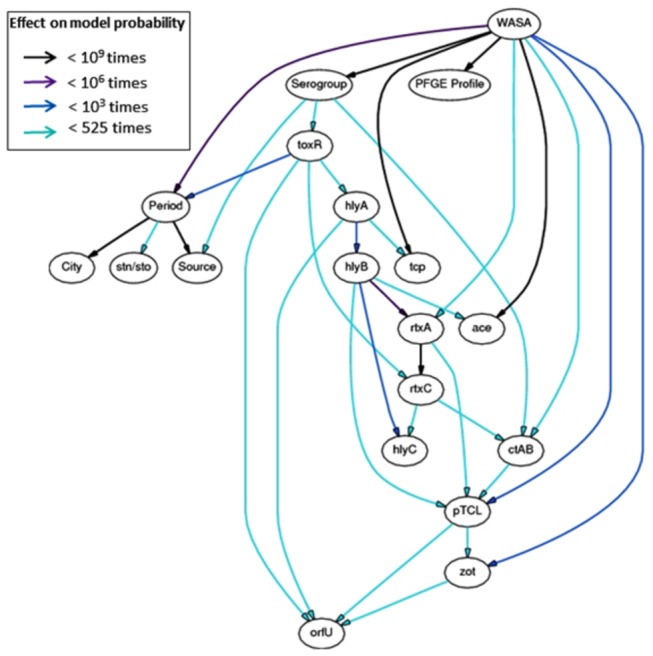
Dependency model of multivariate data from strains. Bayesian network representing conditional probabilities of variables that were available for the strains. Arcs are colored according to the impact in the posterior probability of the model when the arc is removed. The network represents the end result of the evaluation of 4.5 * 10^7^ different topologies, in which the last 1.4 * 10^7^ evaluations did not yield a better model. The network was constructed using the online B-Course software [42].

The possible correlation between the presence of genes, PFGE profiles and other data available from the strains (Table **S1**) was estimated using a statistical learning tool to build a best-topology Bayesian Network (Figure **7**). The constructed model shows significant relations which can be logically explained, adding to the knowledge about the population of environmental *V. cholerae* from the Amazon in the periods that were studied (strains used as references were not included in the constructed model). Strong dependency was observed between period, city, and source which were influenced by the composition of the samples we had available for this study. The PFGE profile was not important in determining any of the variables, while the *WASA-1* prophage correlates strongly with: (i) the definition of serogroups, (ii) PFGE profiles, and (iii) *tcpA* and *ace* (removing arcs reduce the model probability >10^9^ times). The *WASA-1* prophage was also the most significant determinant of *pTLC*, and *zot*. Furthermore, strong arcs were also observed between *hlyB* and *rtxA*, and between *rtxA* and *rtxC*. 

## Discussion

The composition and population structure of environmental *V. cholerae* strains from the Amazon region have not yet been the subject of extensive studies. Though previous works have included clinical and environmental O1 and NAG strains from this region isolated in different periods [51-53], the present study is, to our knowledge, the first time that strains spanning a large time and space interval—temporarily including pre-epidemic, epidemic, post-epidemic isolates—have been evaluated. Previous studies of environmental strains from other regions of Brazil have shown several patterns regarding the presence and distribution of virulence-related genes and population diversity accessed through several strain-typing methods [33,51-53]. 

Though different studies have used different gene sets to characterize the virulence-related genotype of *V. cholerae* strains in Brazil, some of the common genes and genomic elements can be compared. In a study by Theophilo et al., 2006 [51], the presence of *ctxA*, *zot*, *ace*, and *tcpA* was investigated in clinical and environmental strains from different states of Brazil. These strains were isolated between 1991 and 2000. *ctxA* was found in clinical and environmental NAG strains from Northeastern states of Brazil, but not in any strains from the Amazon. *zot*, on the other hand, was the most common gene among these strains, and was amplified individually in some of the strains, and was also co-amplified with *ctxA*, *tcpA*, and/or *ace* in some of the other strains. This reveals a great diversity in the integration and profile of the CTXφ genes among these strains. This diversity has also been observed in other countries, where *ctxAB* was found in strains that were negative for tcpA [34]. *tcpA* encodes the receptor for the integration of the *CTXφ* [45].

NAG *V. cholerae* strains positive for *ctxA* but negative for *zot*, both of which are positive or negative for *tcpA* have also been reported from environmental sources from the Brazilian state of São Paulo [33]. Among these strains—similar to the environmental strains from the Amazon here reported—*hlyA* and *toxR* were the most frequently amplified genes. The same study evaluated O1 strains from clinical and environmental sources isolated during the epidemic period in São Paulo. In contrast to the environmental O1s from the Amazon, all of these strains were positive for *ctxA*, *zot*, and *tcp*. Significant diversity was found among the CTXφ genes and *tcpA* in a more recent study of environmental O1 isolates from the aquatic basin of state of Pernambuco [54]. O1 strains with partial sets of CTXφ genes with and without tcpA were identified, similar to what we found among the environmental O1 isolates from the Amazon.

In our study, all of the epidemic O1 strains from clinical sources were positive for all four CTXφ genes and *tcpA*. In the strains from environmental sources, a significant diversity was observed regarding the presence or absence of the above genes, which suggests mechanisms of gene loss and possibly attenuated virulence among these strains. The clinical manifestations of cholera are dependent in many aspects on the products of the CTXφ genes [44], specially the cholera toxin, which is a product of the *ctxAB* cluster. The colonization of the small intestine depends on the products of the tcpA gene [45,47]. Other aspects of the *V. cholerae* virulence program are also related to *ace* and *zot* [55,56]. For this reason, the isolation of strains potentially lacking a partial set of these genes suggests that their virulence could be attenuated. The fact that these strains share similar PFGE profiles with clinical epidemic isolates suggests that they are not different populations that co-circulated with epidemic isolates, but were indeed originally epidemic strains that lost genes while struggling in the environment. Nevertheless, PCR evidence is not sufficient to conclusively determine the absence of these genes. Complete genome sequences, which our group is currently analyzing, will provide further evidence about what genomic events occurred in the non-amplified regions of these strains. 

Two important issues can be distinguished from comparative analysis of the strains in our study. The first is the virulence profile and virulence potential that the strains exhibit regarding their gene composition. The second is the relation between different populations, which can suggest patterns regarding their origin and dispersion. Genome restriction-pattern analysis through PFGE is limited to elucidating recent clonal divergence and generally fails in reconstructing the genetic relation between populations that diverged over a longer period of time [57]. Since identity and diversity are measured according to similar genome fragment sizes that are within two restriction sites, both cryptic similarity and an overestimation of diversity are possible. Knowledge of the strains and the use of other methods are invaluable to identify and correct possible inconsistencies.

In our study, all of the toxigenic O1 El Tor strains were clustered within three groups which were the most similar to each other (Figure **4**). One of these groups was shared with other O1 El Tor strains that were *ctxAB* negative but harbored other CTXφ genes, suggesting gene-loss events rather than a misclustering of a separated group. All of these strains were positive for the *WASA-1* prophage, which reinforces their identity as epidemic strains [30]. One of the O1 strains (V.735), which was isolated during the epidemic period, was *ctxAB* negative and clustered with other environmental NAGs rather than with the three aforementioned mentioned O1 El Tor clusters. This strain was positive for the *WASA-1* prophage, and harbored the *tcpA* gene. A closer look at its band profile shows that only two of its bands were not shared by any other strains of the El Tor clusters, strongly suggesting that this was likewise originally an epidemic strain.

Attempts to draw conclusions regarding the abundance or true diversity of the *V. cholerae* population in the Brazilian Amazon during the studied period is limited because only a few strains were available. Modeling the interrelations of the multivariate data through a Bayesian network proved to be an efficient manner of structuring the data in a way that facilitates expert analysis and conclusions. In a sense, the data was reduced to a smaller set of interrelations exhibiting quantified uncertainty between them. In this analysis, the *WASA-1* prophage, when compared to other genes that were investigated, exhibited greater importance in discriminating several of the variables that were evaluated. 

To consider an environmental strain as being of epidemic origin, at least three of the following conditions were considered: (i) it was isolated during an epidemic period; (ii) it is of the O1 serotype; (iii) it harbors CTXφ genes and *tcpA*; and (iv) it has a similar PFGE pattern as clinical isolates from the epidemic. None of these conditions, alone, would be enough to determine the others. For instance, in epidemic periods indigenous and epidemic strains were isolated from the environment; O1 isolates from non-epidemic lineages were isolated from clinical and environmental sources; strains with CTXφ genes were found among NAGs, and strains from epidemic origin that were negative for *tcpA* were also identified and; one strain with three of the aforementioned conditions clustered with indigenous strains rather than isolates from the epidemic. All of the strains that harbor the *WASA-1* prophage genes meet three or all four of the conditions, while all strains that are negative for this element do not. Evidence so far shows that investigating the presence of the *WASA-1* prophage among environmental isolates from the Amazon is the best way of determining if the strain is from an epidemic origin or not. Similarly consistent genetic markers from epidemic strains of other places have been identified and suggested for use as molecular identifiers of the strains in clinical and environmental sources. The VCA phage from the toxigenic US Gulf Coast strain and the CTX variant from the Haitian strains are examples of these molecular markers [28,58].

## Supporting Information

Table S1 **Detailed profiles of strains used in this study.**
(PDF)

Table S2 **List of primers used in this study.**
(PDF)

Figure S1 **PFGE cladogram of strains used in this study.**
(PDF)
