# Supplementary material for: Population and Genetic Study of Vibrio cholerae from the Amazon Environment Confirms that the WASA-1 Prophage Is the Main Marker of the Epidemic Strain that Circulated in the Region
Source: PLoS One. 2013 Nov 26;8(11):e81372. doi: 10.1371/journal.pone.0081372 (PMC3841125; doi:10.1371/journal.pone.0081372)
Supplement: Table S1 — Detailed profiles of strains used in this study. (PDF) [file pone.0081372.s001.pdf]

Key	Country	State	City	Site	Source	Year	Serogroup	*ctxAB*	*zot*	*ace*	*orfU*	*rtxA*	*rtxC*	*toxR*	*tcpA*	*pTLC*	*snt/sto*	*hlyA*	*hlyC*	*hlyB*	WASA	Genotype	Pulsetype (DICE/UPGMA)
**N16961 El Tor**	Bangladesh				Human stool sample	1971	O1	+	+	+	+	+	+	+	+	+	-	+	+	+	-	37	1
**121 El Tor**	India				Human stool sample	1973	O1	+	+	+	+	+	+	+	+	+	-	+	+	+	-	37	3
**V.490**	Brazil	Para	Belem		Wastewater	1977	NAG	-	-	-	-	+	+	-	-	-	-	+	+	+	-	1	8
**V.491**	Brazil	Para	Belem		Wastewater	1977	NAG	-	-	-	-	+	+	-	-	-	-	+	+	+	-	1	8
**V.492**	Brazil	Para	Belem		Wastewater	1977	NAG	-	-	-	-	+	+	-	-	-	-	+	+	+	-	1	8
**V.493**	Brazil	Para	Belem		Wastewater	1977	NAG	-	-	-	-	+	-	+	-	-	-	+	+	+	-	2	10
**V.494**	Brazil	Para	Belem		Wastewater	1977	NAG	-	-	-	-	+	-	+	-	-	-	+	+	+	-	2	10
**V.495**	Brazil	Para	Belem		Wastewater	1977	NAG	-	-	-	-	+	+	+	-	-	-	+	+	+	-	3	11
**V.496**	Brazil	Para	Belem		Wastewater	1977	NAG	-	-	-	-	+	+	+	-	-	-	+	+	+	-	3	11
**V.500**	Brazil	Para	Belem		Wastewater	1977	NAG	-	-	-	-	+	+	+	-	-	-	+	+	+	-	3	11
**V.505**	Brazil	Para	Belem		Wastewater	1977	NAG	-	-	-	-	+	+	-	-	-	-	+	+	+	-	1	14
**V.504**	Brazil	Para	Belem		Wastewater	1977	NAG	-	-	-	-	+	+	-	-	+	-	+	+	+	-	6	14
**V.497**	Brazil	Para	Belem		Wastewater	1977	NAG	-	-	-	-	-	-	-	-	-	-	-	+	+	-	4	16
**V.501**	Brazil	Para	Belem		Wastewater	1977	NAG	-	-	-	-	+	+	-	-	-	-	+	+	+	-	1	17
**V.502**	Brazil	Para	Belem		Wastewater	1977	NAG	-	-	-	-	+	+	-	-	-	-	+	+	+	-	1	17
**V.503**	Brazil	Para	Belem		Wastewater	1977	NAG	-	-	-	-	+	+	-	-	+	+	+	+	+	-	5	17
**V.525**	Brazil	Para	Belem		Wastewater	1978	NAG	-	-	-	-	-	-	-	-	-	-	+	-	-	-	12	7
**V.520**	Brazil	Para	Belem		Wastewater	1978	NAG	-	-	-	-	-	-	+	-	-	-	+	-	-	-	8	8
**V.521**	Brazil	Para	Belem		Wastewater	1978	NAG	-	-	-	-	-	-	-	-	-	-	-	-	-	-	9	8
**V.524**	Brazil	Para	Belem		Wastewater	1978	NAG	-	-	-	-	+	+	-	-	-	+	+	+	+	-	11	8
**V.526**	Brazil	Para	Belem		Wastewater	1978	NAG	-	-	-	-	+	+	-	-	-	-	-	-	+	-	13	11
**V.518**	Brazil	Para	Belem		Wastewater	1978	NAG	-	-	-	-	+	+	+	-	-	-	+	+	+	-	3	13
**V.517**	Brazil	Para	Belem		Wastewater	1978	NAG	-	-	-	-	-	-	-	-	-	-	-	+	-	-	7	13
**V.522**	Brazil	Para	Belem		Wastewater	1978	NAG	-	-	-	-	-	+	+	-	-	-	-	-	-	-	10	14
**V.1232**	Brazil	Para	Belem		Wastewater	1979	NAG	-	-	-	-	+	+	+	-	-	+	+	+	+	-	14	13
**V.530**	Brazil	Para	Belem		Wastewater	1980	NAG	-	-	-	-	+	+	+	-	-	-	+	+	+	-	3	13
**V.531**	Brazil	Para	Belem		Wastewater	1980	NAG	-	-	-	-	+	+	-	-	-	-	+	+	+	-	1	17
**V.532**	Brazil	Para	Belem		Wastewater	1980	NAG	-	-	-	-	+	+	-	-	-	-	+	+	+	-	1	17
**V.529**	Brazil	Para	Belem		Wastewater	1980	NAG	-	-	-	-	+	+	-	-	-	-	+	-	-	-	15	17
**V.528**	Brazil	Para	Belem		Wastewater	1980	NAG	-	-	-	-	-	-	-	-	-	-	-	-	-	-	9	18
**V.540**	Brazil	Para	Belem		Wastewater	1981	NAG	-	-	-	-	+	+	-	-	-	-	+	-	+	-	17	12
**V.1233**	Brazil	Para	Belem		Wastewater	1981	NAG	-	-	-	-	+	+	+	-	-	-	+	+	+	-	3	13
**V.539**	Brazil	Para	Belem		Wastewater	1981	NAG	-	-	-	-	+	+	-	-	-	-	+	+	-	-	16	16
**V.541**	Brazil	Para	Belem		Wastewater	1981	NAG	-	-	-	-	+	+	-	-	-	-	+	+	+	-	1	17
**V.542**	Brazil	Para	Belem		Wastewater	1981	NAG	-	-	-	-	+	+	-	-	-	-	+	+	+	-	1	17
**200 classical**	Pakistan				Human stool sample	1986	O1	+	+	+	+	-	-	+	+	+	-	+	+	+	-	49	6
**V.1101**	Brazil	Amapa	Macapa	Hotel	Wastewater	1991	NAG	-	-	+	-	+	+	+	-	-	-	+	+	+	-	18	4
**V.1103**	Brazil	Amapa	Macapa	Hotel	Wastewater	1991	NAG	-	-	-	-	+	+	+	-	-	-	+	+	+	-	3	9
**V.1097**	Brazil	Amapa	Macapa	Fazendinha Beach	Superficial river water	1991	NAG	-	-	-	-	+	+	+	-	-	-	+	+	+	-	3	11
**V.1102**	Brazil	Amapa	Macapa	Hotel	Wastewater	1991	NAG	-	-	-	-	-	-	-	-	-	-	-	-	+	-	19	12
**V.1099**	Brazil	Amapa	Macapa	Beiradao River	Superficial river water	1991	NAG	-	-	-	-	+	+	-	-	-	-	+	+	+	-	1	14
**V.1098**	Brazil	Amapa	Macapa	Araxa Beach	Superficial river water	1991	NAG	-	-	-	-	+	+	+	-	-	-	+	+	+	-	3	14
**V.1100**	Brazil	Amapa	Macapa	Beiradinho River	Superficial river water	1991	NAG	-	-	-	-	-	-	-	-	-	-	-	-	-	-	9	20
**V.621**	Brazil	Para	Barcarena	Porto da Balsa	Superficial river water	1991	O1	+	+	+	+	+	+	+	+	+	-	+	+	+	+	36	1
**V.613**	Brazil	Amapa	Macapa	Amazon River	Superficial river water	1991	O1	+	+	+	+	+	+	+	+	+	-	+	+	+	+	36	1
**VC512**	Brazil	Amazonas			Human stool sample	1991	O1	+	+	+	+	+	+	+	+	+	-	+	+	+	+	36	1
**V.595**	Brazil	Para	Belem	Guajara Bay	Superficial river water	1991	O1	+	+	+	+	-	+	+	+	+	-	+	+	+	+	40	1
**V.1124**	Brazil	Amapa	Macapa	Cachoeira do Navio River	Superficial river water	1991	O1	+	+	+	-	-	-	-	+	+	-	+	-	+	+	38	2
**V.1125**	Brazil	Amapa	Macapa		Wastewater	1991	O1	-	+	+	+	-	-	+	-	+	-	+	+	+	+	39	2
**V.1142**	Brazil	Amapa	Macapa	Fish (Locariidae)	Fish	1992	NAG	-	-	-	-	+	+	-	-	-	-	-	+	+	-	20	4
**V.1145**	Brazil	Amapa	Macapa	Fish (Hoplias sp.)	Fish	1992	NAG	-	-	+	-	+	+	+	-	-	-	+	+	+	-	18	8
**V.1143**	Brazil	Amapa	Macapa		Wastewater	1992	NAG	-	-	-	+	+	+	+	-	+	-	+	+	+	-	21	8
**V.1140**	Brazil	Amapa	Macapa		Wastewater	1992	NAG	-	-	-	-	+	+	+	-	-	-	+	+	+	-	3	9
**V.409**	Brazil	Para	Belem	Icoaraci	Superficial stream water	1992	NAG	-	-	+	-	+	+	+	-	-	-	+	+	+	-	18	9
**V.1141**	Brazil	Amapa	Macapa	Fish (Cichlid)	Fish	1992	NAG	-	-	-	-	+	+	+	-	-	-	+	+	+	-	3	12
**V.411**	Brazil	Para	Belem	Icoaraci	Superficial stream water	1992	NAG	-	-	-	-	-	-	-	-	-	-	-	-	-	-	9	14
**V.410**	Brazil	Para	Belem	Icoaraci	Superficial stream water	1992	NAG	-	-	-	-	+	+	+	-	-	-	+	+	+	-	3	22
**V.408**	Brazil	Para	Belem	Icoaraci	Superficial stream water	1992	NAG	-	-	-	-	-	-	+	-	-	-	-	-	-	-	22	22
**V.729**	Brazil	Para	Belem	Baia do Sol	Superficial river water	1992	O1	+	+	+	+	+	+	+	+	+	-	+	+	+	+	36	1
**VC514**	Brazil	Para	Belem		Human stool sample	1992	O1	+	+	+	+	+	+	+	+	+	-	+	+	+	+	36	1
**V.733**	Brazil	Amazonas	Tabatinga	Solimoes River	Superficial river water	1992	O1	+	+	+	+	+	+	+	+	+	-	+	+	+	+	36	1
**VC513**	Brazil	Amazonas			Human stool sample	1992	O1	+	+	+	+	+	+	+	+	+	-	+	+	+	+	36	1
**V.725**	Brazil	Para	Belem	Guajara Bay	Superficial river water	1992	O1	+	+	+	+	-	+	+	+	+	-	+	+	+	+	40	1
**V.734**	Brazil	Amazonas	Tabatinga	Solimoes River	Superficial river water	1992	O1	+	+	+	+	+	-	+	+	+	-	+	+	+	+	41	1
**V.736**	Brazil	Amazonas	Tabatinga	Solimoes River	Superficial river water	1992	O1	+	-	+	-	-	-	+	+	+	-	+	+	+	+	43	2
**V.737**	Brazil	Amazonas	Tabatinga	Solimoes River	Superficial river water	1992	O1	+	+	+	+	+	+	+	-	+	-	+	+	+	+	44	2
**L34**	Brazil	Amazonas	Tabatinga		Human stool sample	1992	O1	-	-	-	-	+	+	+	-	-	-	+	+	+	-	3	8
**V.735**	Brazil	Amazonas	Tabatinga	Solimoes River	Superficial river water	1992	O1	-	-	+	-	+	-	+	+	-	-	+	+	+	+	42	9
**V.1172**	Brazil	Acre	Rio Branco	COHAB	Wastewater	1993	NAG	-	-	-	-	+	+	-	-	-	-	-	+	+	-	20	9
**V.1169**	Brazil	Acre	Rio Branco	Bus station waste	Wastewater	1993	NAG	-	-	-	-	+	+	-	-	-	-	+	+	+	-	1	10
**V.1171**	Brazil	Acre	Rio Branco	Pretorio Stream	Superficial stream water	1993	NAG	-	-	-	-	+	-	+	-	-	-	+	+	+	-	2	10
**V.1173**	Brazil	Acre	Rio Branco	Baixa Verde	Wastewater	1993	NAG	-	-	-	-	-	+	-	-	-	-	-	-	-	-	24	10
**V.773**	Brazil	Para	Belem	Guama River	Superficial river water	1993	NAG	-	-	-	-	-	-	-	-	-	-	-	+	-	-	7	17
**V.1168**	Brazil	Acre	Rio Branco	Purus-Peru River	Superficial river water	1993	NAG	-	-	-	-	-	-	-	-	-	-	+	+	+	-	23	17
**V.412**	Brazil	Para	Belem	UNA	Wastewater	1993	O1	+	+	+	+	+	-	+	+	+	-	+	-	+	+	45	1
**V.780**	Brazil	Acre	Rio Branco	Acre River	Superficial river water	1993	O1	+	+	+	+	+	+	+	+	+	-	+	+	+	+	36	3
**V.778**	Brazil	Acre	Santa Rosa	Purus River	Superficial river water	1993	O1	+	+	+	-	-	+	+	-	+	-	-	+	+	+	36	3
**V.797**	Brazil	Amapa	Macapa	P. Socorro Channel	Superficial stream water	1994	NAG	-	-	-	-	+	+	+	-	-	-	+	+	+	-	3	5
**V.428**	Brazil	Para	Belem	Tucunduba Stream	Superficial stream water	1994	NAG	-	-	-	-	+	+	+	-	-	-	+	+	+	-	3	7
**V.803**	Brazil	Amapa	Santana	Santana Port	Superficial river water	1994	NAG	-	-	-	-	-	-	-	-	-	-	+	+	+	-	23	7
**V.802**	Brazil	Amapa	Macapa	Fortaleza Stream	Superficial stream water	1994	NAG	-	-	-	-	-	-	+	-	-	-	+	+	+	-	25	7
**V.423**	Brazil	Para	Belem	Guajara Bay	Superficial river water	1994	NAG	-	-	-	-	+	+	-	-	-	-	+	+	+	-	1	10
**V.799**	Brazil	Amapa	Macapa	Araxa Beach	Superficial river water	1994	NAG	-	-	+	-	+	+	-	-	-	-	-	+	+	-	24	12
**V.420**	Brazil	Para	Belem	UNA	Wastewater	1994	NAG	-	-	-	-	+	+	+	-	-	-	+	+	+	-	3	15
**V.798**	Brazil	Amapa	Macapa	Araxa Beach	Superficial river water	1994	NAG	-	-	-	-	-	-	-	-	-	-	-	-	-	-	9	18
**V.800**	Brazil	Amapa	Macapa	Jandia Channel	Superficial stream water	1994	NAG	-	-	-	-	-	-	-	-	-	-	-	-	-	-	9	20
**V.796**	Brazil	Amapa	Oiapoque	Oiapoque River	Superficial river water	1994	NAG	-	-	-	-	-	-	-	-	-	-	+	+	+	-	23	20
**V.482**	Brazil	Para	Belem	UNA	Wastewater	1994	O1	+	+	+	+	+	+	+	+	+	-	+	+	+	+	36	1
**VC816**	Brazil	Para			Human stool sample	1994	O1	+	+	+	+	+	+	+	+	+	-	+	+	+	+	36	1
**V.548**	Brazil	Para	Belem	UNA	Wastewater	1994	O1	+	+	+	+	+	+	+	+	+	-	+	+	+	+	36	2

**V.485**	Brazil	Para	Belem	UNA	Wastewater	1994	O1	+	-	-	-	+	+	+	+	-	-	+	-	-	+	46	2
**V.543**	Brazil	Para	Belem	UNA	Wastewater	1994	O1	+	+	-	+	+	+	+	+	+	-	+	+	+	+	47	2
**V.794**	Brazil	Amapa	Oiapoque	Oiapoque River	Superficial river water	1994	O1	-	+	+	+	-	-	+	+	+	-	+	+	+	+	48	2
**V.452**	Brazil	Para	Barcarena		Superficial river water	1995	NAG	-	-	-	-	+	+	+	-	-	-	+	+	+	-	3	9
**V.454**	Brazil	Para	Barcarena		Superficial river water	1995	NAG	-	-	-	-	+	+	+	-	-	-	-	+	+	-	26	9
**V.457**	Brazil	Para	Belem	UNA	Wastewater	1995	NAG	-	-	-	-	+	-	+	-	-	-	+	+	+	-	2	10
**V.465**	Brazil	Para	Belem	Guama River	Superficial river water	1996	NAG	-	-	-	-	+	+	-	-	-	-	+	+	+	-	1	22
**V.472**	Brazil	Para	Belem	Guama River	Superficial river water	1996	NAG	-	-	-	-	+	+	-	-	-	-	-	+	+	-	20	23
**V.946**	Brazil	Para	Belem	Mosqueiro	Superficial river water	1997	NAG	-	-	-	-	+	+	+	-	-	-	+	+	+	-	3	5
**V.951**	Brazil	Para	Belem	Mosqueiro	Superficial river water	1997	NAG	-	-	-	-	+	+	+	-	-	-	+	+	+	-	3	5
**V.874**	Brazil	Para	Maruda		Copepod	1997	NAG	-	-	-	-	+	+	+	-	-	+	+	+	+	-	14	6
**V.877**	Brazil	Para	Maruda		Copepod	1997	NAG	-	-	-	-	+	+	+	-	-	+	+	+	+	-	14	14
**V.907**	Brazil	Para	Belem	Tucunduba Stream	Superficial stream water	1998	NAG	-	-	-	-	+	+	+	-	-	-	+	+	+	-	3	17
**V.916**	Brazil	Para	Belem	Guama River	Superficial river water	1998	NAG	-	-	-	-	+	+	-	-	-	-	+	+	+	-	1	21
**V.913**	Brazil	Para	Belem	Guama River	Superficial river water	1998	NAG	-	-	-	-	-	-	-	-	-	-	+	+	+	-	23	21
**V.1090**	Brazil	Para	Belem	Guama River	Superficial river water	1999	NAG	-	-	-	-	+	+	+	-	-	-	+	+	+	-	3	5
**V.1018**	Brazil	Para	Belem	Guajara Bay	Superficial river water	1999	NAG	-	-	-	-	+	+	+	-	-	-	+	+	+	-	3	7
**V.1013**	Brazil	Para	Belem	Tucunduba Stream	Superficial stream water	1999	NAG	-	-	-	-	-	-	+	-	-	-	+	+	+	-	25	7
**V.1052**	Brazil	Para	Belem	Guama River	Superficial river water	1999	NAG	-	-	-	-	+	+	+	-	-	+	+	+	+	-	14	8
**V.1015**	Brazil	Para	Belem	Guama River	Superficial river water	1999	NAG	-	-	-	-	+	+	-	-	-	-	+	+	+	-	1	12
**V.1032**	Brazil	Para	Belem	Guajara Bay	Superficial river water	1999	NAG	-	-	-	-	-	-	+	-	-	+	+	+	+	-	27	12
**LMA519-4**	Brazil	Para	Belem	Tucunduba Stream	Superficial stream water	2000	NAG	-	-	+	-	+	+	+	-	-	+	+	+	+	-	28	5
**LMA541-3**	Brazil	Para	Belem	Guama River	Superficial river water	2000	NAG	-	-	-	-	+	-	+	-	-	-	+	-	+	-	29	10
**LMA575-2**	Brazil	Para	Belem	Guajara Bay	Superficial river water	2000	NAG	-	-	-	-	+	-	+	-	-	-	-	+	+	-	30	12
**LMA840-2**	Brazil	Para	Belem	Guama River	Superficial river water	2001	NAG	-	-	-	-	+	+	+	-	-	-	+	+	+	-	3	8
**LMA750-1**	Brazil	Para	Belem	Guajara Bay	Superficial river water	2001	NAG	-	+	-	-	+	+	+	-	-	-	+	+	+	-	31	9
**LMA841-4**	Brazil	Para	Belem	Tucunduba Stream	Superficial stream water	2001	NAG	-	-	-	-	+	-	+	-	-	-	-	-	-	-	33	14
**LMA820-1**	Brazil	Para	Belem	UNA	Wastewater	2001	NAG	-	-	-	-	+	-	+	-	-	+	-	-	+	-	32	15
**LMA1814-1**	Brazil	Para	Belem		Superficial stream water	2002	NAG	-	-	-	-	+	+	+	-	-	-	+	+	+	-	3	11
**LMA1809-1**	Brazil	Para	Belem	Guajara Bay	Superficial river water	2002	NAG	-	-	-	-	+	+	+	-	-	-	+	+	+	-	3	12
**LMA2215-9**	Brazil	Para	Belem	Guajara Bay	Superficial river water	2003	NAG	-	-	-	-	+	+	+	-	-	+	+	+	+	-	14	6
**LMA2159-1**	Brazil	Para	Belem	Guama River	Superficial river water	2003	NAG	-	-	-	-	+	+	+	-	-	-	+	+	+	-	3	10
**LMA2217-9**	Brazil	Para	Belem	Tucunduba Stream	Superficial stream water	2003	NAG	-	-	-	-	+	+	+	-	-	-	+	+	+	-	3	11
**LMA2495-1**	Brazil	Para	Belem	Guama River	Superficial river water	2004	NAG	-	-	-	-	+	-	+	-	-	+	+	+	+	-	34	5
**LMA2314-6**	Brazil	Para	Belem	Tucunduba Stream	Superficial stream water	2004	NAG	-	-	-	-	+	+	+	-	-	-	+	+	+	-	3	10
**LMA2638-1**	Brazil	Para	Belem	Guajara Bay	Superficial river water	2005	NAG	-	-	-	-	+	+	+	-	-	-	+	+	+	-	3	7
**LMA2688-9**	Brazil	Para	Belem	UNA	Wastewater	2005	NAG	-	-	-	-	+	+	+	-	-	-	+	+	+	-	3	11
**LMA2712-4**	Brazil	Para	Belem	Tucunduba Stream	Superficial stream water	2005	NAG	-	-	-	-	+	+	+	-	-	-	+	+	+	-	3	12
**LMA3460-1**	Brazil	Para	Belem	Guajara Bay	Superficial river water	2006	NAG	-	-	-	-	+	+	+	-	-	-	+	+	+	-	3	7
**LMA3603-2**	Brazil	Para	Belem	Tucunduba Stream	Superficial stream water	2006	NAG	-	-	-	-	+	+	+	-	-	-	+	+	+	-	3	8
**LMA3501-4**	Brazil	Para	Belem	Guama River	Superficial river water	2006	NAG	-	-	-	-	+	+	+	-	+	-	+	+	+	-	35	12
**LMA3598-1**	Brazil	Para	Belem	UNA	Wastewater	2006	NAG	-	-	-	-	+	+	+	-	+	-	+	+	+	-	35	19
**LMA3984-4**	Brazil	Para	Belem	Tucunduba Stream	Superficial stream water	2007	O1	-	-	-	-	+	+	+	-	-	-	+	+	+	-	3	6
