# Supplementary material for: Population and Genetic Study of Vibrio cholerae from the Amazon Environment Confirms that the WASA-1 Prophage Is the Main Marker of the Epidemic Strain that Circulated in the Region
Source: PLoS One. 2013 Nov 26;8(11):e81372. doi: 10.1371/journal.pone.0081372 (PMC3841125; doi:10.1371/journal.pone.0081372)
Supplement: Table S2 — List of primers used in this study. (PDF) [file pone.0081372.s002.pdf]

PRIMERS USED IN THIS STUDY

Gene	Primer pair	Hybridization Temperature
ompW	CACCAAGAAGGTGACTTTATTGTG<br>GAACTTATAACCACCCGCG	55 ^{0}C
ctxAB	GCAGTCAGGTGGTCTTATTGC<br>TCCAGATATGCAATCCTCAG	52 ^{0}C
zot	TAAACCTTGAACGCATAG<br>CGCCCATAGACCACGATA	47 ^{0}C
ace	TAAGGATGTGCTTATGATGGACACCC<br>GGTGATGAATAAAGATACTCATAGG	53 ^{0}C
orfU	GCTGCATTTATCCTTATTGCC<br>TGTAACCAAACGCCTCGAC	55 ^{0}C
rtxA	CTGAATATGAGTGGGTGACTTACG<br>GTGTATTGTTCGATATCCGCTACG	56 ^{0}C
rtxC	CGACGAAGATCATTGACGAC<br>CATCGTCGTTATGTGGTTGC	56 ^{0}C
toxR	TCGGATTAGGACACAACTC<br>CTGCGAGGGGAAGTAAGAC	50 ^{0}C
tcp	GGTGACTTTGTGTGGTTAAATG<br>CCATAATCCGACACCTTG	52 ^{0}C
pTLC	CTACGCCAGACCATCAAACAG<br>CACCCTCACTCAGGTTTTGC	56 ^{0}C
stn/sto	GAGAAACCTATTCATTGCA<br>GCAAGCTGGATTGCAAC	48 ^{0}C
hlyA	GAGCGTAATGCGAAGAATGC<br>GAGTCAGGTTTTGGTTACAGG	53 ^{0}C
hlyC	AAATCCGCCACTTCTCTTC<br>AATCAAAGCCACCAAGCC	54 ^{0}C
hlyB	CAAGCCTTCGCCAATAAC<br>CCACTTTTTTCCCTTCACC	54 ^{0}C
WASA-1	CTTCCGGCGTCATTGGGCGT<br>CGGTGTAGCGGGTGACGTGG	60 ^{0}C
WASA-1	AGCGTGTTCACGGTCAGCCT	60 ^{0}C

	GCGCGTCAACGCTCGGTAGA	
WASA-1	CCGCTTGAATCAACCACGGTCACA<br><br>CGAGGGAAACATTGGGGCGGG	60 ^{0}C
