# Supplementary figures and images for: Population and Genetic Study of Vibrio cholerae from the Amazon Environment Confirms that the WASA-1 Prophage Is the Main Marker of the Epidemic Strain that Circulated in the Region
Source: PLoS One. 2013 Nov 26;8(11):e81372. doi: 10.1371/journal.pone.0081372 (PMC3841125; doi:10.1371/journal.pone.0081372)

SAMPLE PFGE PROFILES

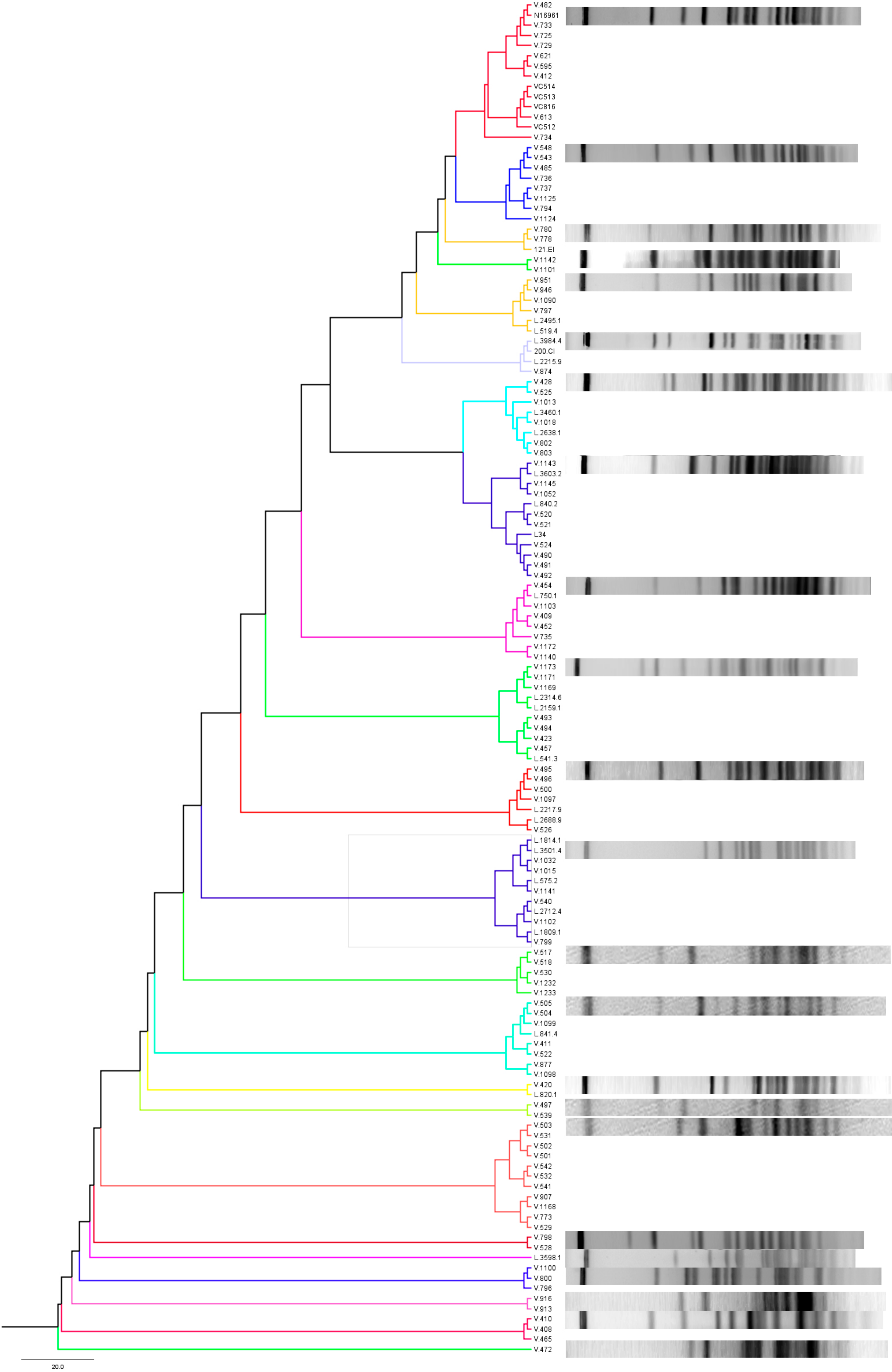

Supplement: Figure S1 — PFGE cladogram of strains used in this study. (PDF) [file pone.0081372.s003.pdf]
